# Adverse events risk associated with anti-VEGFR agents in the treatment of advanced nonsmall-cell lung cancer: A meta-analysis

**DOI:** 10.1097/MD.0000000000003752

**Published:** 2016-12-02

**Authors:** Biao Gu, WenChuang Gao, HongJun Chu, Jian Gao, Zhi Fu, Hui Ding, JunJie Lv, QingQuan Wu

**Affiliations:** a Department of Thoracic Surgery, Huai’an First People's Hospital, Nanjing Medical University; b Department of Thoracic Surgery, Lian Shui People's Hospital, Lianshui, Huai’an; c Department of Thoracic Surgery, Nantong Third People's Hospital, Nantong University, Nantong; d Department of Analysis, Huai’an First People's Hospital, Nanjing Medical University, Huai’an, Jiangsu.

**Keywords:** adverse events, angiogenesis inhibitor, anti-VEGFR agents, meta-analysis, nonsmall-cell lung cancer, safety

## Abstract

To perform this meta-analysis, we investigated the risk of the most clinically relevant adverse events related to antivascular endothelial growth factor receptor (VEGFR) agents in advanced nonsmall-cell lung cancer (NSCLC).

A comprehensive literature search for studies published up to October 2015 was performed. Prospective randomized controlled phase II/III clinical trials that comparing therapy with or without anti-VEGFR agents for advanced NSCLC were included for analysis. Summary relative risk (RR) and 95% confidence intervals (CIs) were calculated using random effects or fixed effects according to the heterogeneity among included trials.

A total of 11,701 patients from 18 clinical trials were included for analysis. Pooled RR showed that the use of anti-VEGFR agents significantly increased the risk of developing hypertension (RR 4.71, 95% CI 3.29–6.73, *P* < 0.001) and fatal adverse events (RR 1.33, 95% CI 1.12–1.58, *P* = 0.001). No statistically significant differences were found for gastrointestinal (GI) perforation (*P* = 0.41), arterial or venous thromboembolic events (*P* = 0.49 and *P* = 0.16, respectively), or hemorrhagic events (*P* = 0.81). Sensitive analysis indicated that the significance estimate of pooled RR of fatal adverse event (FAEs) was not significantly influenced by omitting any single study.

The use of anti-VEGFR agents in advanced NSCLC does significantly increase the risk of hypertension and fatal adverse events, but not for arterial or venous thromboembolic events, GI perforation, or hemorrhagic events.

## Abstract 2

Supplemental Digital Content is available in the text

## 1 Introduction

Lung cancer is one of the most common diagnosed malignant tumors throughout the world but also the leading cause of cancer death in males in 2008. Among females, it is the fourth-most commonly diagnosed cancer and the second leading cause of cancer death.^[1,2]^ Approximately 85% of lung cancer cases are nonsmall-cell lung cancer (NSCLC), with 65% to 75% of them having locally advanced or metastatic disease.^[3]^ Outcomes for patients with advanced NSCLC remain poor, with the 5-year survival rate being <4% .^[4,5]^ Clearly, it is necessary to develop novel agents to achieve greater survival benefits for advanced NSCLC patients.

During the past decades, a better understanding of the molecular events involved in the tumor angiogenesis of cancers has led to the development of new-targeted agents. Basic research has shown that angiogenesis is mainly driven by vascular epithelial growth factor (VEGF), thus angiogenesis inhibitors targeting the VEGF signal pathway is a potentially effective strategy for the treatment of advanced NSCLC.^[6,7]^ Bevacizumab, a humanized monoclonal antibody targeting VEGF-A, has been approved for use in advanced NSCLC cancer due to its potential survival benefits.^[8,9]^ Recently, several novel angiogenesis inhibitors targeting the VEGF receptors (VEGFR), such as sorafenib, vandetanib, nintedanib, ramucirumab, and cediranib, are currently being under investigation.^[10–14]^ Nintedanib, a small molecule tyrosine kinase inhibitor that targets receptors for VEGF, has been also approved for use in combination with docetaxel as second-line treatment for locally advanced, metastatic, or locally recurrent NSCLC.^[15,16]^ Another humanized VEGFR-2 monoclonal antibody ramucirumab has also been approved as second-line treatment for advanced NSCLC.^[17]^ In addition, 2 recent meta-analyses also demonstrated that the use of anti-VEGFR agents in advanced NSCLC significantly improved objective response rate and progression-free survival when compared with controls.^[18,19]^ Therefore, the use of these drugs is expected to increase in the near future, and it would be useful for clinicians to clearly know the severe adverse events (AEs) related to anti-VEGFR agents in the treatment of advanced NSCLC. However, to our best knowledge, there is no specific systematic review and meta-analysis focusing on the clinically relevant toxicities associated with anti-VEGFR agents in these patients. We therefore conduct this comprehensive meta-analysis of randomized controlled trials to assess the overall risk of severe AEs related to anti-VEGFR agents in the treatment of advanced NSCLC.

## 2 Materials and methods

### 2.1 Study design

We conducted this meta-analysis adhere to the Preferred Reporting Items for Systematic Review and Meta-analyses statements (supplemental Table 1). This study did not involve human subjects, so informed consent was not required. In addition, no approval was required from any institutional review board.

### 2.2 Selection of studies

The Cochrane Central Register of Controlled Trials (CENTRAL), PubMed (up to December 2015), and Web of Science (up to December 2015) databases were searched for articles using “VEGFR-TKIs,” “angiogenesis inhibitor,” “sorafenib,” “sunitinib,” “vandetanib,” “axitinib,” “pazopanib,” “cediranib,” “nintedanib,” “motesanib,” “ramucirumab,” “regorafenib,” “anti-VEGFR agents,” “non-small-cell lung cancer,” “prospective,” “phase II/III,” “randomized controlled trial,” and “humans.” To assess the relationship between the use of anti-VEGFR agents and clinically significant adverse events, we studied AEs classified as grade ≥3 by the NCI-CTC.^[23]^ Clinical trials that met the following requirements were included: prospective randomized controlled phase II and III trial in advanced NSCLC patients, participants assigned to treatment with or without anti-VEGFR agents, and events or event rate and sample size available regarding adverse outcomes of interest (grade ≥3 AEs of arterial thromboembolic events [ATEs], venous thromboembolic events [VTEs], hypertension, GI perforation, hemorrhagic events, and fatal adverse events [FAEs]) and sample size.

### 2.3 Data extraction

Two investigators independently performed data extraction. Any discrepancies between reviewers were resolved by consensus. If reviewers suspected an overlap of cohorts in a report, they contacted the corresponding author for clarification; we excluded studies with a clear overlap. The following information was recorded for each study: first author's name, year of publication, study phase, treatment line, number of patients enrolled, treatment regimens, median age, median progression-free survival, number of patients available for analysis, number of events of the following adverse events: grade ≥3 AEs of ATEs, VTEs, hypertension, GI perforation, hemorrhagic events, and FAEs.

### 2.4 Statistical analysis

For each trial, data on FAEs and severe AEs (grade 3 or 4) associated with anti-VEGFR agents were extracted and pooled to calculate relative risks (RR) with 95% confidence interval (CI). The χ^2^-based Q statistic test was used for the assessment of the between-study heterogeneity and it was considered significant when *P*_heterogeneity_ < 0.05 or *I*^2^ > 50%.^[20]^ If heterogeneity existed, data were analyzed using a random effects model. In the absence of heterogeneity, a fixed effects model was used. An estimate of potential publication bias was carried out using Begg and Egger tests.^[21,22]^ The results of the meta-analysis were reported as classic forest plots. The Jadad scale was used to assess the quality of included trials based on the reporting of the studies’ methods and results.^[23]^ All statistical analysis was carried out with comprehensive meta-analysis software version 2.0 (Biostat, Englewood, NJ), using two-sided *P* value.

## 3 Results

### 3.1 Search results

**Figure 1:**
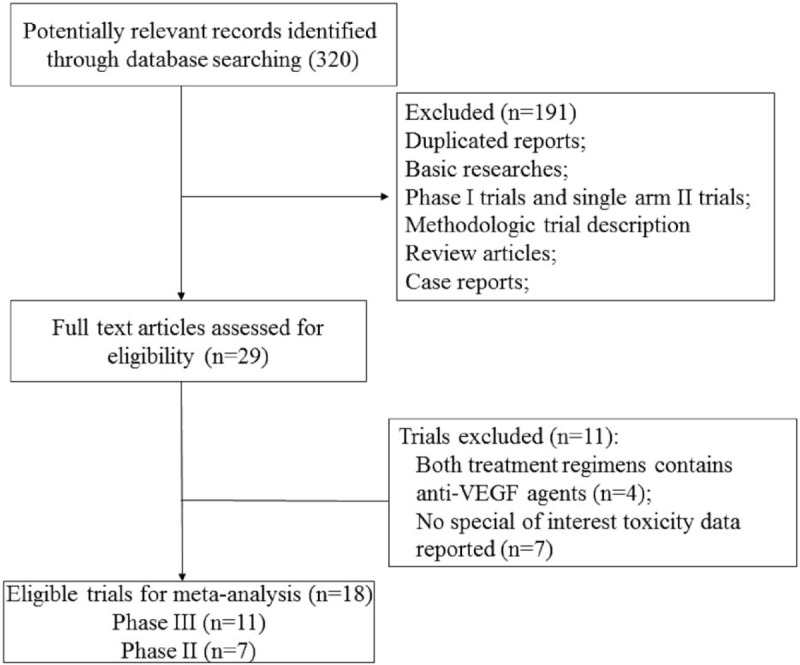
Studies eligible for inclusion in the meta-analysis.

We identified a total of 320 relevant studies according to the search strategy, and 29 reports were retrieved for full-text review. In the review, 11 articles were excluded. Finally, 18 trials that met the inclusion criteria were included for analysis.^[10–14,24–36]^Figure 1 provided the flow chart. The main characteristics of the included trials were presented in Table 1. The sample sizes of the studies ranged from 124 to 1391 patients (total, 11,701). The quality of the included trials was high. Thirteen trials were double-blinded, randomized, placebo-controlled trials, and had a Jadad score of 5. The other 5 trials had a Jadad score of 3. Table 2 describes the distribution of the number of patients and associated reported AEs in each of the treatment arms for each of the included studies.

**Table 1:**
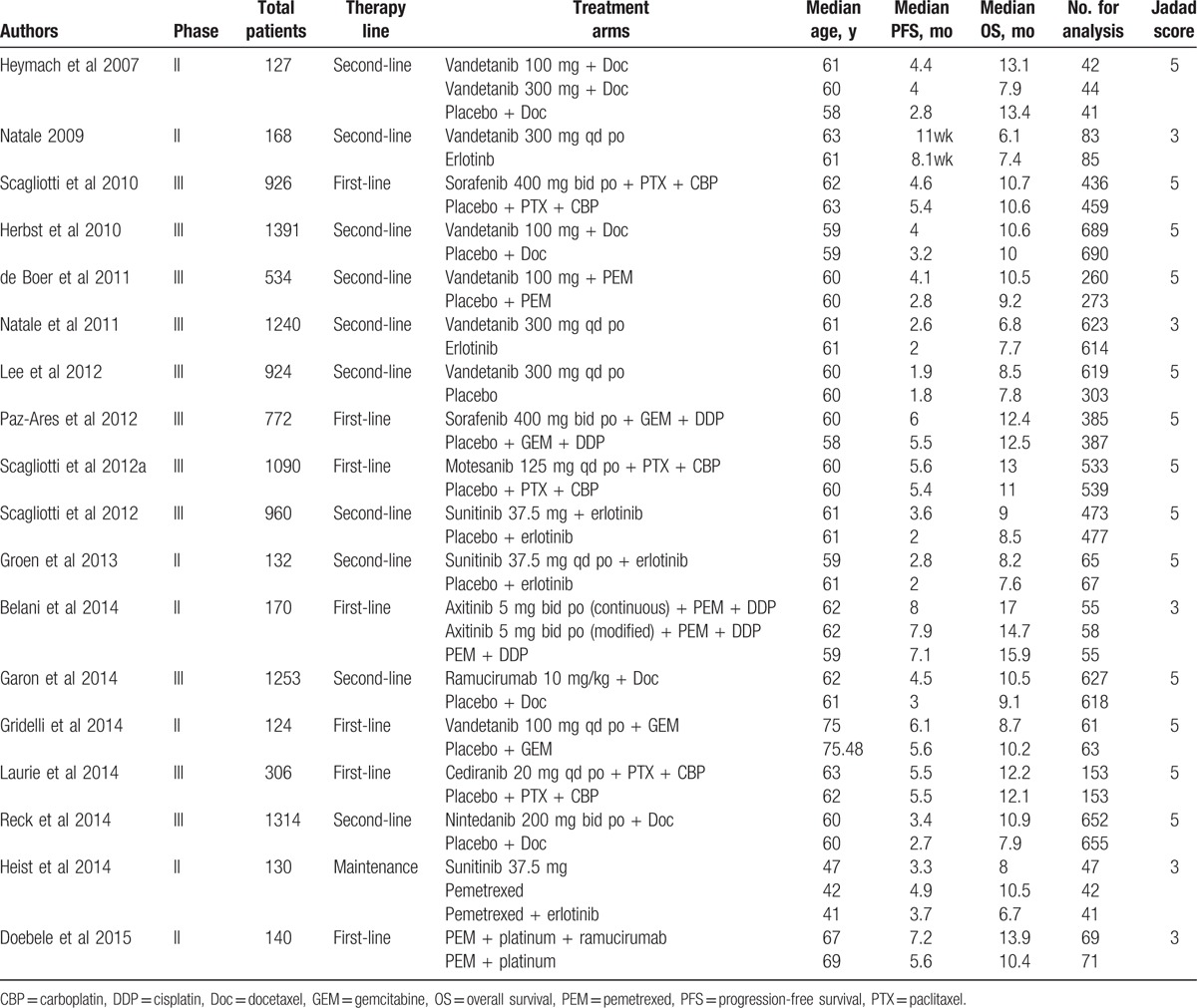
Baseline characteristics of 18 randomized controlled trials for analysis.

**Table 2:**
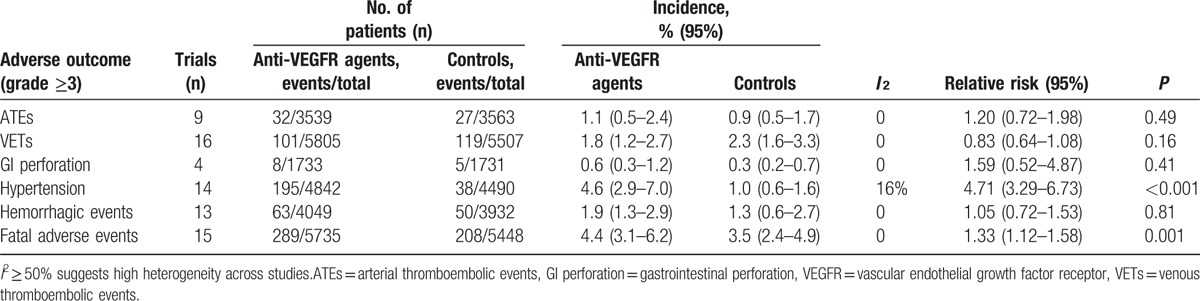
Relative risk of adverse outcomes for clinical trials included in the meta-analysis.

### 3.2 Heterogeneity

No observed heterogeneity for ATEs, VTEs, GI perforation, hypertension, hemorrhagic events, or FAEs was found (Table 2). We thus used fixed effects model to pool the risk of severe AEs related to anti-VEGFR agents.

### 3.3 AEs reported in trials and pooled effects

#### 3.3.1 Arterial and venous thromboembolic events

**Figure 2:**
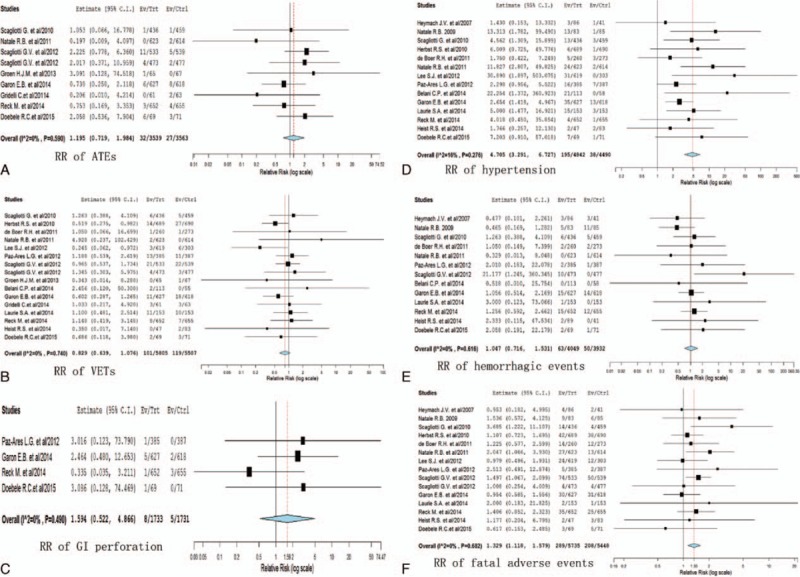
Risk of severe adverse outcomes associated with anti-VEGFR treatment compared with control treatment [All graphs show RR for each study and summary RR obtained for (A) ATEs, (B) VTEs, (C) GI perforation, (D) hypertension, (E) hemorrhagic events, (F) fatal adverse events]. The size of squares corresponds to the weight of the study in the meta-analysis. The diamond plot represents the overall results of the included trials. ATEs = arterial thromboembolic events, GI = gastrointestinal, RR = risk ratio, VEGFR = vascular endothelial growth factor receptor, VTEs = venous thromboembolic events.

A total of 59 patients with ATEs was reported, 32 (1.1%) in anti-VEGFR arms and 27 (0.9%) in control arms. The RR among the included studies ranged from 0.197 to 3.091. And the pooled results did not find an increased risk of ATEs associated with anti-VEGFR agents using a fixed effects model (RR = 1.20; 95% CI 0.72–1.98; *P* = 0.49, Fig. 2A).

A total of 16 trials reported VTEs data. The pooled incidence of VTEs in anti-VEGFR agent arms was relatively lower than that in control arms (1.8% vs 2.3%). The pooled RR showed that the use of anti-VEGFR agents did not increase the risk of VTEs compared with controls (RR = 0.83, 95% CI 0.64–1.08, *P* = 0.16, Fig. 2B).

### 3.4 GI perforation

Only 4 trials reported GI perforation data with 8 (0.6%) patients in anti-VEGFR agents arms, and 5 (0.3%) in control arms. We did not observe increased risk of GI perforation with anti-VEGFR agents-containing regimens using a fixed effects model (RR = 1.59, 95% CI 0.52–4.87, *P* = 0.41, Fig. 2C).

### 3.5 Hypertension

Fourteen trials reported hypertension data with a total of 233 patients experiencing grade ≥3 hypertension. The pooled incidence of severe hypertension was more frequent (4.6%) in anti-VEGFR agents group than those in the control group (1.0%). The pooled RR was 4.71 (95% CI 3.29–6.73, *P* < 0.001) using a fixed effects model (Fig. 2D).

### 3.6 Hemorrhagic events

A total of 113 severe hemorrhagic events were reported in the trials; 63 (1.9%) in anti-VEGFR agent arms and 50 (1.3%) in control arms. This conferred an overall RR of developing hemorrhagic events of 1.05 (95% CI 0.72–1.53, *P* = 0.81) (Fig. 2E).

### 3.7 Grade 5 toxicities

**Figure 3:**
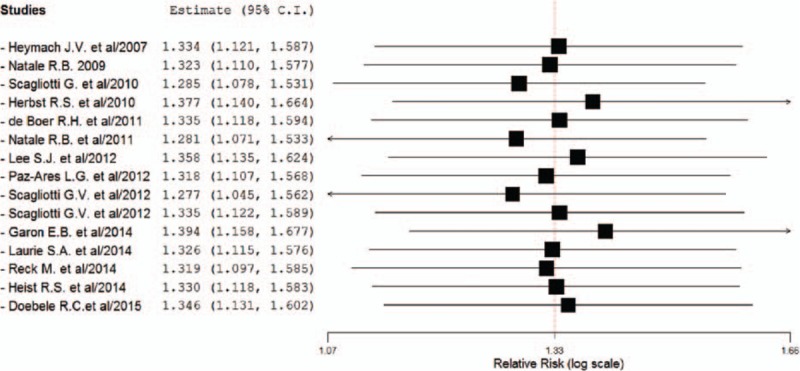
Meta-analysis of fatal adverse events associated with anti-VEGFR agents versus control: “leave-one-out” sensitivity analysis. VEGFR = vascular endothelial growth factor receptor.

A total of 289 (4.4%) grade 5 AEs were observed in the anti-VEGFR agent group and 208 (3.5%) in the control group. This confers a pooled RR of developing grade 5 events of 1.33 (95% CI 1.12–1.58, *P* = 0.001 (Fig. 2F). We also did sensitivity analysis to examine the stability and reliability of pooled RRs by sequential omission of individual studies. The results indicated that the significance estimate of pooled RR of FAEs was not significantly influenced by omitting any single study (Fig. 3).

### 3.8 Publication bias

No publication bias was detected for the AEs studied except for hypertension by either the Begg or Egger tests (Begg test, *P* = 0.10; Egger test, *P* = 0.04, Table 3).

**Table 3:**
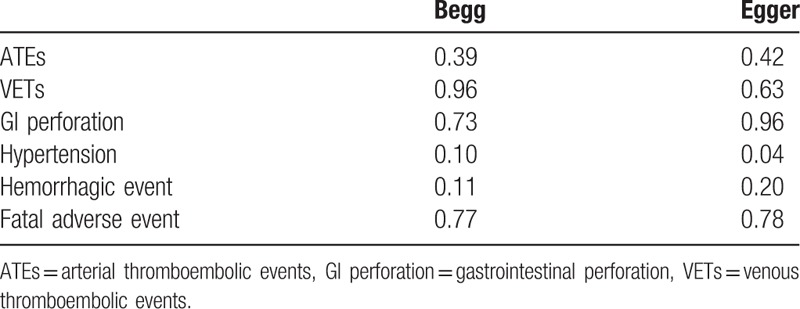
Publication bias Begg and Egger test (*P* value).

## 4 Discussion

Angiogenesis, especially VEGF signal pathway, plays a pivotal role in tumor growth, progression, and metastasis.^[37,38]^ Thus, the VEGF signal pathway has been targeted as a therapeutic option for solid tumors including NSCLC. However, VEGF plays multiple roles in physiologic processes, and thus its inhibition could have potentially serious systemic consequences. Although previous researches have shown that anti-VEGFR agents significantly increases the risk of developing anti-VEGF adverse events, including hypertension,^[39–41]^ hemorrhage,^[42,43]^ proteinuria,^[44,45]^ gastrointestinal perforation, ^[46]^ congestive heart failure,^[47–49]^ and thromboembolic events.^[50–53]^, the risk of these adverse events in advanced NSCLC remains unknown. Our study includes a total of 11,701 patients to investigate the relationship between those AEs with anti-VEGFR agent use. The pooled results show that the use of anti-VEGFR agents is associated with a significantly increased risk of developing grade ≥3 hypertension and FAEs in comparison with controls, whereas no significant relationship is found between anti-VEGFR agents use and risk of GI perforation, ATEs or VTEs, or hemorrhagic events.

The study of hypertension events shows the highest RR with 4.71, which is consistent with the previously published meta-analyses.^[39–41]^ As we know, severe hypertension including hypertensive crisis may cause significant cardiovascular damage with a possible life-threatening consequence, and limit the use of anti-VEGFR agents. Therefore, it is particularly important for all clinicians to monitor and treat hypertension in a timely manner and appropriately to prevent long-term complications from toxicities.

Several previous meta-analyses have indicated a significantly increased risk of FAEs associated with anti-VEGFR agents in solid tumors,^[54–56]^ but the risk of FAEs with these agents in advanced NSCLC remains undetermined. In the study conducted by Sivendran et al,^[55]^ the authors found that there was no significant increased risk of FAEs with anti-VEGFR agents in NSCLC (RR 1.88, 95% CI 0.96–3.68, *P* = 0.07), whereas an increased risk of FAEs (OR 2.37, 95% CI 1.19–4.73, *P* = 0.01) was observed in another study conducted by Hong et al.^[54]^ In our study focusing on NSCLC patients, grade 5 adverse events are rare and more frequent in the anti-VEGFR agents arm than in the control arm (4.4% vs 3.5%, respectively), and the incidence of FAEs with anti-VEGFR agents is the same as previously reported by Sivendran et al^[55]^ (4.4%). Our pooled results with the largest sample size demonstrate that the use of anti-VEGFR agents in advanced NSCLC significantly increase the risk of FAEs (RR 1.33, 95% CI 1.12–1.58, *P* = 0.001). As angiogenesis inhibitors find more clinical applications and are used to treat a more heterogeneous patient population than those found in clinical trials, clinicians should be aware of the risk of FAEs when treating NSCLC patients with these drugs.

We then assess the risk of vascular events with anti-VEGFR agents in NSCLC patients. Our study does not observe a statistically significant increase of ATEs or VTEs with anti-VEGFR agent use in NSCLC patients. We also do not find the use of these drugs is associated with an increased risk of GI perforation (RR = 1.15, *P* = 0.41). GI perforation seems to be less frequent in NSCLC than in other tumor types, suggesting tumor-dependent mechanisms. In addition, we do not find a significant increased risk of hemorrhagic events with the use of anti-VEGFR agent in NSCLC patients (RR = 1.05, *P* = 0.81).

We have to acknowledge a number of limitations in the present study. First, this is a meta-analysis at study level, confounding variables at the patient level could not be incorporated into the analysis. Second, although adverse events are prospectively collected for each individual study, this analysis remains a retrospective research that is subject to the method deficiencies of the included trials. We minimize the likelihood of bias by strictly selecting randomized clinical trials with direct comparison with and without anti-VEGFR agents before the analysis. Finally, as in all meta-analyses, our results may be biased as a result of potential publication bias. However, a funnel plot evaluation for the severe AEs does not indicate publication bias except for hypertension.

## 5 Conclusions

In conclusion, the use of anti-VEGFR agents in advanced NSCLC did significantly increase the risk of hypertension and FAEs, but not for ATEs or VTEs, GI perforation, or hemorrhagic events. Clinicians should be aware of these risks and perform regular monitoring of NSCLC patients receiving anti-VEGFR agents.

## Supplementary Material

Supplemental Digital Content
